# Intracellular Renin Disrupts Chemical Communication between Heart Cells. Pathophysiological Implications

**DOI:** 10.3389/fendo.2014.00238

**Published:** 2015-01-22

**Authors:** Walmor C. De Mello

**Affiliations:** ^1^School of Medicine, University of Puerto Rico, San Juan, PR, USA

**Keywords:** chemical communication, heart cell, intracellular renin, angiotensinogen, heart function

## Abstract

**Highlights**

- Intracellular renin disrupts chemical communication in the heart
- Angiotensinogen enhances the effect of renin
- Intracellular enalaprilat reduces significantly the effect of renin
- Intracellular renin increases the inward calcium current
- Harmful versus beneficial effect during myocardial infarction

The influence of intracellular renin on the process of chemical communication between cardiac cells was investigated in cell pairs isolated from the left ventricle of adult Wistar Kyoto rats. The enzyme together with Lucifer yellow CH was dialyzed into one cell of the pair using the whole cell clamp technique. The diffusion of the dye in the dialyzed and in non-dialyzed cell was followed by measuring the intensity of fluorescence in both cells as a function of time. The results indicated that; (1) under normal conditions, Lucifer Yellow flows from cell to cell through gap junctions; (2) the intracellular dialysis of renin (100 nM) disrupts chemical communication – an effect enhanced by simultaneous administration of angiotensinogen (100 nM); (3) enalaprilat (10^−9^ M) administered to the cytosol together with renin reduced drastically the uncoupling action of the enzyme; (4) aliskiren (10^−8^ M) inhibited the effect of renin on chemical communication; (5) the possible role of intracellular renin independently of angiotensin II (Ang II) was evaluated including the increase of the inward calcium current elicited by the enzyme and the possible role of oxidative stress on the disruption of cell communication; (6) the possible harmful versus the beneficial effect of intracellular renin during myocardial infarction was discussed; (7) the present results indicate that intracellular renin due to internalization or *in situ* synthesis causes a severe impairment of chemical communication in the heart resulting in derangement of metabolic cooperation with serious consequences for heart function.

## Introduction

The circulating renin–angiotensin system (RAS) regulates blood volume and blood pressure through the release of renin, which cleaves Ang I from angiotensinogen followed by the conversion of Ang I into Ang II by the angiotensin converting enzyme (ACE). Evidence is now available that renin is also present in extrarenal tissues including the heart and adrenal gland (1, 2). Although renin uptake from plasma is an important source of cardiac renin (3), a renin transcript located exclusively intracellularly and overexpressed during myocardial infarct in rats (4, 5) indicates a role for cytosolic renin in post-ischemic repair processes (1). Evidence is available that the cytosolic renin is functionally active and has effects opposite to those of circulating renin (1). In transgenic rats overexpressing cytosolic renin, for instance, the blood pressure is normal and the plasma levels of renin are low (1), revealing that the function of cytosolic renin is different from that of circulating renin.

Previous studies indicated that intracellular renin reduces the electrical coupling of heart cells – an effect enhanced by intracellular angiotensinogen (2). Since enalaprilat reduced drastically the effect of intracellular renin on gap junction conductance (2), the conclusion is that the activation of an intracellular RAS plays an important role in the regulation of cell-to-cell communication by changing the gap junction conductance (6).

The role of gap junctions, however, is not limited to the spread of ions and electrical current between heart cells but it is also involved in the exchange of chemical information between cells. Small molecules like amino acids, nucleotides, and second messengers, for instance, flow easily from cell to cell establishing an important example of metabolic cooperation (7). Indeed, when donor cells (BHK) prelabeled with 3H-uridine and washed containing labeled uridine nucleotides are co-cultured with unlabeled cells, transference of labeled material to unlabeled cells is seen when intercellular junctions are established between them (8).

Recent studies indicated that high glucose disrupts the chemical communication between cardiac cells – a phenomenon dependent on PKC activation and on the enhanced intracellular levels of Ang II elicited by high glucose (9). No information is available if intracellular renin alters the chemical communication between cardiac cells. In the present work, this problem was investigated in cell pairs isolated from the ventricle of adult rats.

## Materials and Methods

Normal adult Wistar Kyoto rats were used. The animals were kept in the animal house at constant temperature (24°C) and humidity following the recommendations of NIH. Animals were kept on a normal laboratory animal diet and given tap water *ad libitum*. The animals were anesthetized with 43 mg/kg of ketamine plus 5 mg/kg of xylazine and the heart was removed with the animals under deep anesthesia. All animal procedures were approved by the IACUC.

### Cell isolation procedure

The heart was removed and immediately perfused with normal Krebs solution containing: (in millimoles): NaCl – 136.5; KCl – 5.4; CaCl_2_ – 1.8; MgCl_2_ – 0.53; NaH_2_PO_4_ – 0.3; NaHCO_3_ – 11.9; glucose – 5.5; HEPES – 5, pH adjusted to 7.3. After 20 min, a Ca-free solution containing 0.4% collagenase (Worthington Biochemical Corp.) was recirculated through the heart for 1 h. The collagenase solution was washed out with 100 ml of recovery solution containing (millimoles): taurine 10; oxalic acid 10; glutamic acid 70; KCl 25; KH_2_PO_4_ 10; glucose 10; pH 7.4. All solutions were oxygenated with 100% O_2_. Ventricles were minced (1–2 mm thick slices) and the resulting solution was agitated gently and the suspension was filtered. The filtrate was centrifuged for 4 min at 22 × *g*. The cell pellets were then resuspended in normal Krebs solution.

### Experimental procedures

All experiments were performed in a small chamber mounted on the stage of an inverted phase-contrast microscope (Diaphot, Nikon). Ventricular cells were placed in a modified cultured dish (volume 0.75 ml) in an open-perfusion microincubator (Model PDMI-2, Medical Systems). Cells were allowed to adhere to the bottom of the chamber for 15 min and were superfused with normal Krebs solution (3 ml/min) that permits a complete change of the bath in <500 ms. A video system made possible to inspect the cells and the pipettes throughout the experiments. The electrical measurements were carried out using the patch-clamp technique in a whole cell configuration with an Axon (model 200B) patch-clamp amplifier and Digidata 1440A (Molecular Devices, CA, USA).

### Measurements of dye coupling

Cell pairs of ventricular myocytes were used. Suction pipettes were pulled from microhematocrit tubing by means of a controlled puller (Narishige, Japan) and filled with a solution with the following composition (millimoles): cesium aspartate 120; NaCl 10; MgCl_2_ 3; tetraethylammonium chloride 20; Na_2_ATP 5; HEPES 5; pH 7.3 containing 4% of Lucifer Yellow CH (mol weight 457 Da). The pipette was attached to one cell of the pair and a gigaohm seal was achieved. The membrane was ruptured by a brief suction allowing the dye to diffuse from the pipette into the cell.

### Measurements of inward calcium current

#### Experimental procedures

All experiments were performed in a small chamber mounted on the stage of an inverted phase-contrast microscope (Diaphot; Nikon). A video system (Diaphot; Nikon) made it possible to inspect the cells and the pipettes throughout the experiments. The electrical measurements were carried out using the patch-clamp technique in a whole cell configuration with an Axon (model 200B) patch-clamp amplifier and Digidata 1440A (Molecular Devices, CA, USA). Series resistance originated from the tips of the micropipettes was compensated electronically at the beginning of the experiment. Membrane currents sampled at 10 kHz were filtered at 2 kHz, and digitized using pClamp10 software (Axon Instruments). The leak currents were digitally subtracted by the P/N method (n$\frac{1}{4}$5–6). Experiments performed without leak subtraction indicated low and stable leak currents. Current/voltage curves were obtained by applying voltage steps in 8-mV increments from a holding potential of −40 mV. All current recordings were obtained after *I*_Ca_ had been stabilized. The peak inward calcium current was measured in the same cell before and after intracellular dialysis of renin (100 nM). Values of calcium currents were normalized for cell capacitance.

### Measurements of oxidative stress

To directly monitor real time reactive oxygen species/reactive nitrogen species (ROS/RNS), a kit including an oxidative stress detection reagent (ENZO Life Sciences, Farmingdale, NY, USA) was used. Cells were exposed to the reagent for 35 min and then renin was dialyzed inside the cell and measurements of fluorescence intensity were made before and after intracellular renin (100 nM) administration using a wide filed fluorescence microscope equipped with standard green (490/525 nm) filter set.

### Drugs

Rat renin was kindly provided by Dr. Jan Danser, Erasmus University, the Netherlands. Angiotensinogen was from Sigma Chemical Co., Saint Louis, MI, USA, and Aliskiren was kindly provided by Novartis Pharmaceuticals. Enalaprilat was from Merck.

### Statistical analysis

Data are expressed as mean ± SEM. Student’s *t*-test was used. Differences were considered significant when *P* < 0.05.

## Results

**Figure 1:**
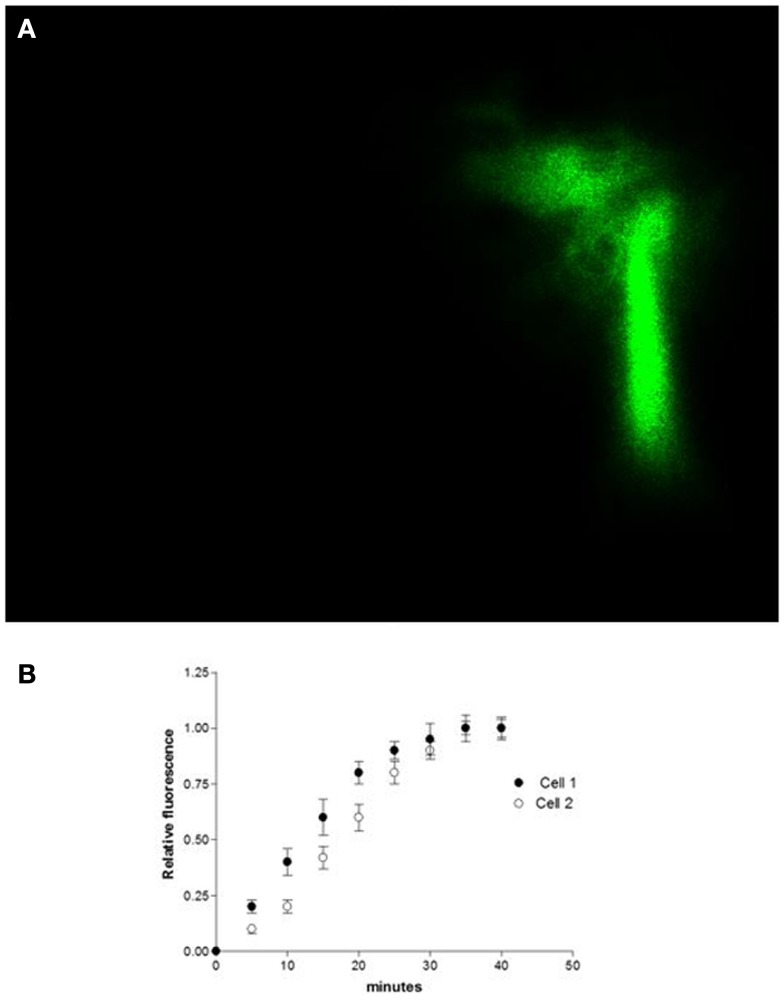
**(A)** shows the intercellular diffusion of Lucifer Yellow CH in single cell pair isolated from the ventricle of adult rat. **(B)** intracellular dialysis of Lucifer Yellow CH into cell 1 and its diffusion to cell 2 through gap junctions. Each point is the average from 29 cell pairs (four animals). Vertical line at each point SEM (*P* < 0.05).

**Figure 2:**
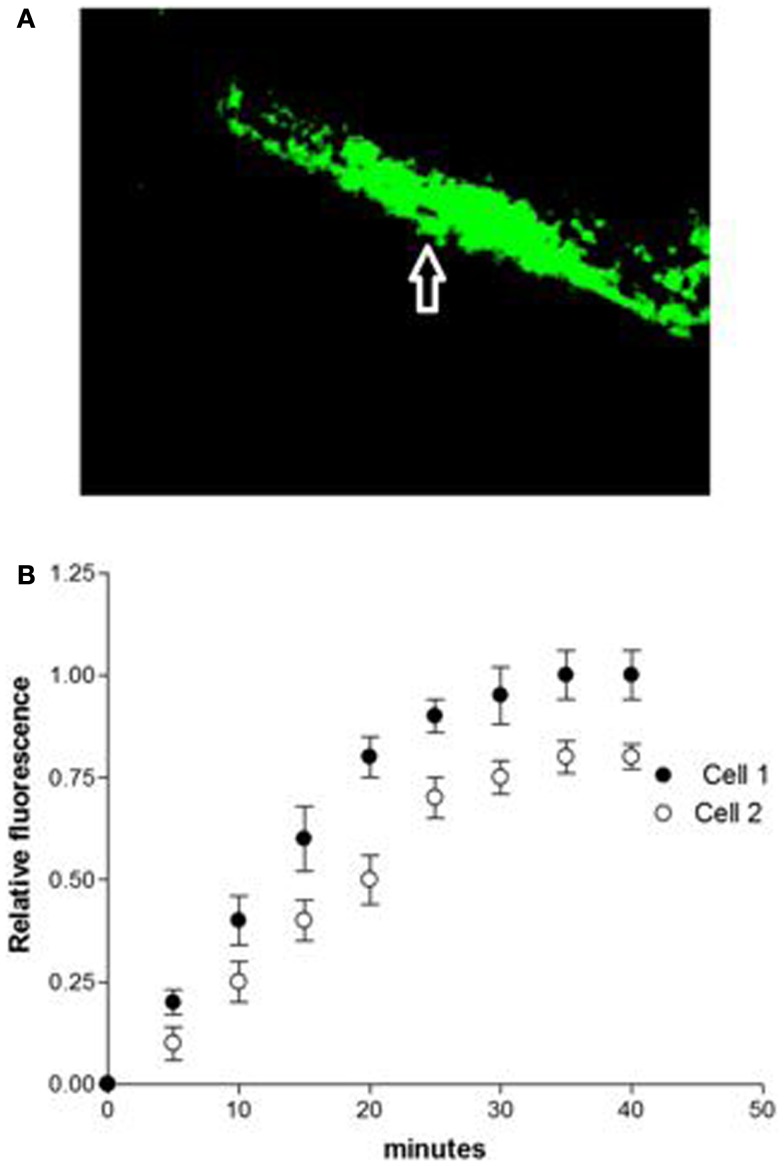
**(A)** Disruption of chemical communication elicited by intracellular renin (100 nM) in single cell pair. **(B)** Disruption of chemical communication cause by intracellular dialysis of renin (100 nM). Each point is the average from 31 cell pairs (five animals). Vertical line at each point SEM (*P* < 0.05).

**Figure 3:**
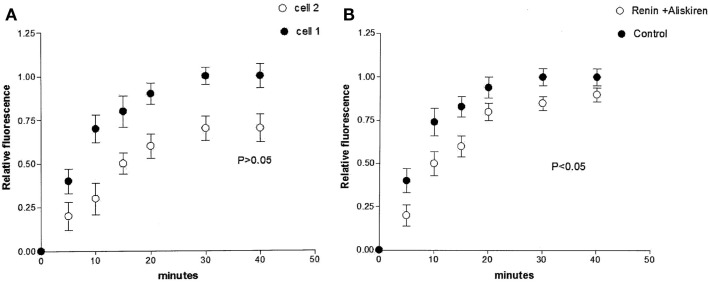
**(A)** Enhanced decoupling action of intracellular renin (100 nM) caused by simultaneous intracellular administration of angiotensinogen (Ao) (100 nM). Each point is the average from 30 cell pairs. Vertical line at each point SEM (*P* < 0.05). **(B)** Inhibition of the effect of intracellular renin elicited by aliskiren (10^−8^ M). Each point is the average from 18 cell pairs. Vertical line at each point SEM (*P* > 0.05).

Studies performed under control conditions, indicated that Lucifer Yellow CH (mol. weight – 457 Da) diffuses initially within the dialyzed cell and then into the adjacent myocyte within 30 s (Figure 1). Since the dye is not able to cross the surface cell membrane (10), it is possible to conclude that the intercellular diffusion of the dye occurs through the gap junctions (10). To investigate the influence of intracellular renin on chemical communication, rat renin (100 nM) was added to the pipette solution containing Lucifer Yellow CH and then dialyzed into one cell of the pair. Measurements of the intensity of fluorescence in each cell of the pair were performed making it possible to follow the spread of the dye between the two cells. The results revealed a drastic reduction of cell communication elicited by renin as shown in Figure 2. Angiotensinogen (Ao) (100 nM) added to the pipette solution together with renin (100 nM) causes a greater decline of chemical communication as seen in Figure 3 while aliskiren (10^−8^ M) inhibited the effect of intracellular renin (see Figure 3B).

**Figure 4:**
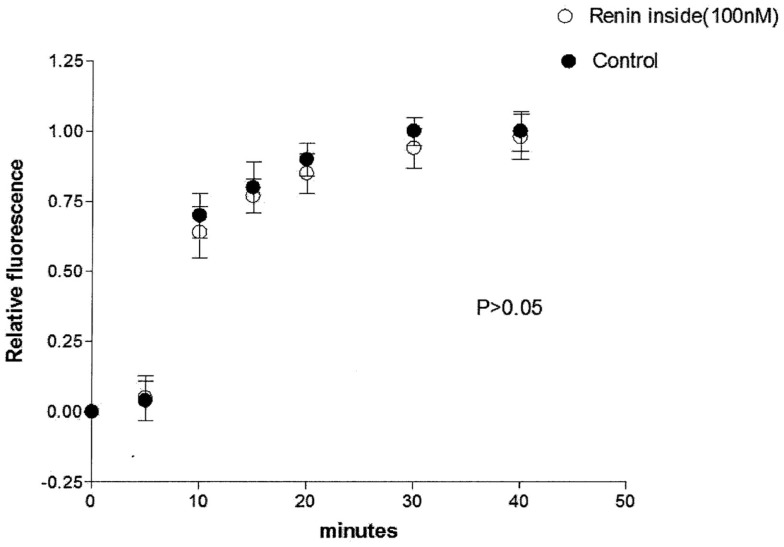
**Lack of influence of intracellular renin (100 nM) on the intracellular diffusion of Lucifer Yellow**. Each point is the average from 16 cells. Vertical line at each point SEM (*P* < 0.05).

The possibility that the diffusion of Lucifer Yellow CH in the cytosol of the dialyzed cell be impaired by intracellular renin with consequent increase of the time needed to reach the non-dialyzed cell was investigated by measuring the time required by the dye to reach the non-dialyzed cell. Results from 16 experiments indicated no change on the intracellular diffusion of Lucifer Yellow CH in cells dialyzed with renin (100 mM) as compared with controls (Figure 4).

### Is renin altering chemical communication independently of Ang II?

#### Influence of enalaprilat

**Figure 5:**
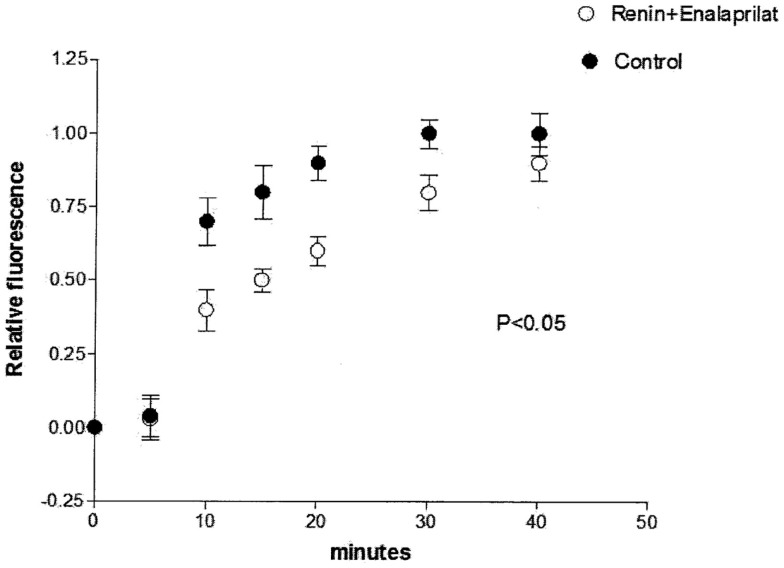
**Partial reestablishing of chemical communication caused by intracellular administration of enalaprilat (10^−9^ M)**. Each point is the average from 31 cell pairs. Vertical line at each point SEM (*P* < 0.05).

To investigate the possibility that intracellular renin is changing the chemical communication independently of Ang II, enalaprilat (10^−9^ M) was added to the pipette solution containing renin (100 nM) plus Lucifer Yellow CH, and measurements of dye coupling were performed. Figure 5 shows that the action of intracellular renin on chemical communication was reduced significantly by intracellular administration of enalaprilat.

#### How influential is intracellular renin on the inward calcium current?

**Figure 6:**
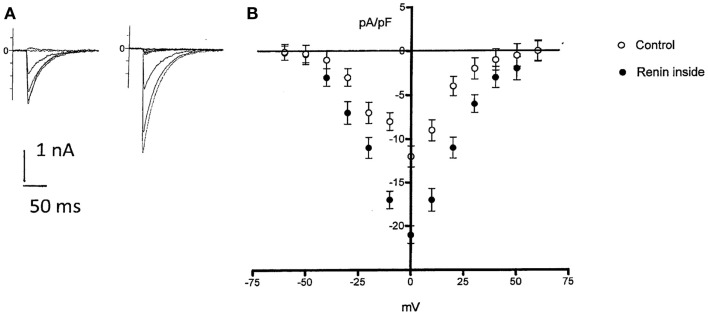
**Top left – effect of intracellular administration of renin (100 nM) on *I*_Ca_ in a single rat myocyte**. Control *I*_Ca_ **(A)** and after 6 min of renin administration **(B)**. Bottom, voltage dependence of *I*_Ca_ in rat myocytes in the absence and after intracellular renin (100 nM) administration. Each point is the average from 28 cells (four animals). Vertical line at each point SEM (*P* < 0.05). Holding potential −40 mV.

Because enalaprilat did not abolish completely the effect of renin, the question remains if part of the effect of renin is independent of Ang II. Since it is known that an increase of the intracellular calcium concentration can cause electrical uncoupling of heart cells (11), it is important to investigate if intracellular renin increases the inward calcium current. To investigate this possibility, the inward calcium current was measured in isolated cardiomyocytes before and after the administration of renin in the cytosol. As shown in Figure 6, intracellular renin (100 nM) increases the density of inward calcium current.

#### Can renin enhance oxidative stress?

**Figure 7:**
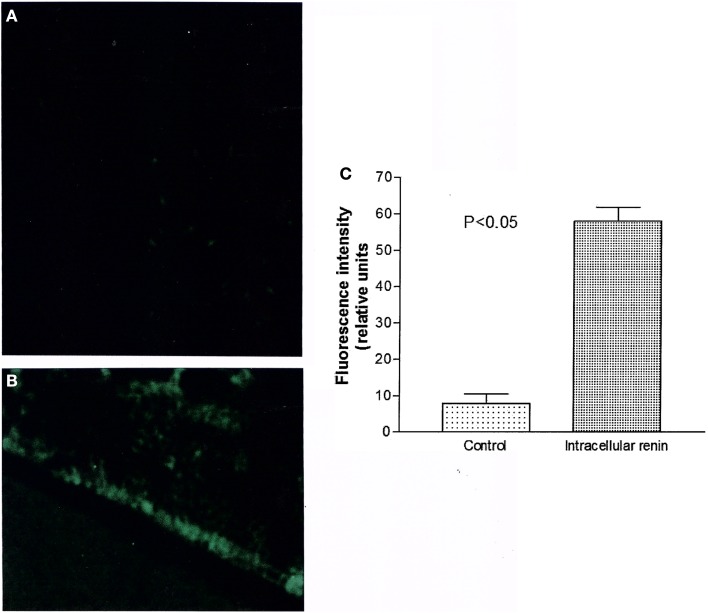
**Measurements of oxidative stress (ROS/RNS) elicited by intracellular renin (100 nM)**. **(A)** Control fluorescence microscopy of single cell in absence of renin; **(B)** Green fluorescence measured in the same cell after 12 min of intracellular renin (100 nM) administration. **(C)** Green fluorescence inside the cell before and after 12 min of intracellular dialysis of renin (100 nM). Each bar is the average from 21 ventricular myocytes (three animals). Vertical line at each bar SEM (*P* < 0.05).

Figure 7 shows the influence of intracellular renin (100 nM) on generation of oxidative stress recorded from ventricular cardiomyocytes recorded after 12 min of renin administration. As it can be seen, the enzyme enhanced the oxidative stress but its effect was slow requiring of at least 10–12 min.

### Influence of intracellular renin on gap junction permeability

A quantitative estimation of the gap junction permeability (*P*_j_) was made using the following equation (12):

$$P_{j}=V_{\mathrm{cell}}∕A_{j}\times K_{j}$$

where *V*_cell_ is the cell volume that is accessible to Lucifer Yellow CH, *A*_j_ is area of the gap junctional membrane, and *K*_j_ is the rate constant of the transjunctional diffusion (12). Morphometric studies performed on ventricular tissues (13) indicate that *A*_j_ contributes to 17% of the cross sectional area of the rat cardiomyocyte (177 mm^2^) and that myofibrils, mitochondria, sarcoplasmic reticulum, and nucleus occupy about 88% of the total cell volume leaving 12% of total cell volume free for the dye diffusion through the cytoplasm. The junctional permeability (*P*_j_) was then calculated for controls and after intracellular dialysis of renin (100 nM), for cells exposed to intracellular renin plus enalaprilat or intracellular renin (100 nM) plus angiotensinogen (Ao) (100 nM). As shown in Table 1, the value of *P*_j_ calculated for the control taken *V*_cell_ as 12% of total cell volume was 2.3 ± 0.04 × 10^−4^ cm/s; *n* = 29 (four animals); and for cells dialyzed with renin (100 nM) was 1 ± 0.05 × 10^−4^ cm/s; *n* = 31 (five animals) (*P* < 0.05). Intracellular administration of angiotensinogen (Ao) (100 nM) together with renin enhanced the decoupling action of renin, because the value of *P*_j_ was reduced to 7.9 ± 0.04 × 10^−5^ *n* = 30 (four animals) (*P* < 0.05). On the other hand, enalaprilat (10^−9^ M) administered to the cytosol together with renin (100 nM) reduced significantly the effect of renin on chemical communication (*P*_j_ = 2.1 ± 0.05 × 10^−4^; *n* = 32 (four animals) (see Table 1).

**Table 1:** **Influence of intracellular renin (100 nM), intracellular renin plus angiotensinogen (Ao) (100 nM) and intracellular renin plus enalaprilat (10^−9^ M) on gap junction permeability (*P*_j_) (cm/s) in rat cardiac myocytes**.

Control		Intracellular renin		Intracellular renin + Ao		Intracellular renin + enalaprilat
2.3 ± 0.04 × 10^−4^ (*n* = 29) (4 animals)		1.1 ± 0.05 × 10^−4^ (*n* = 31) (5 animals)		7.9 ± 0.04 × 10^−5^ (*n* = 30) (4 animals)		2.1 ± 0.05 × 10^−4^ (*n* = 32) (4 animals)
	*P* < 0.05		*P* < 0.05		*P* < 0.05

*V_cell_ taken as 12% of total cell volume for all the experiments*.

## Discussion

The present results indicated for the first time that intracellular renin disrupts the process of chemical communication between cardiac cells what means that the normal exchange of important molecules between myocytes such as cyclic nucleotides, amino acids, neurotransmitters, and other molecules smaller than 1 kDa is inhibited abolishing the metabolic cooperation among heart cells and creating a severe impairment of tissue functioning. The effect of intracellular renin on chemical communication was enhanced when angiotensinogen was dialyzed together with renin inside the cell supporting the view that the formation of Ang II is involved in the decoupling action of intracellular renin. Indeed, enalaprilat administered intracellularly caused a large reduction on the effect of intracellular renin on dye coupling. Previous observations indicated that intracellular Ang II impairs the electrical coupling between cardiac cells through the activation of PKC and consequence phosphorylation of gap junction proteins (6). There is no doubt that renin is present in the heart of adult rats and that the majority of renin is located intracellularly (5). In addition, a transcript of non-secretory renin, which remains inside the heart cell (4) is overexpressed during myocardial infarction (5). The presence of renin, ACE as well as angiotensin II receptors, and angiotensinogen in the cytosol and nuclei (14–17) as well as the identification of a local RAS in mitochondria (18), lead to the conclusion that there is an intracrine RAS (2, 4–11, 14–26).

Although simultaneous administration of angiotensinogen enhanced the effect of intracellular renin on chemical communication, renin alone had a significant effect on dye coupling. Moreover, enalaprilat reduced but not completely abolished the effect of renin, raising the question if part of the impairment of chemical communication elicited by the enzyme is independent of Ang II. Some possibilities can be considered on explaining a possible direct effect of renin on cell communication; (a) intracellular renin increases the intracellular calcium concentration with consequent decrease of junctional permeability. This view is supported by the finding that intracellular renin increased the inward calcium current; (b) the cytosolic renin can interact with the intracellular renin receptor enhancing the expression of several genes (21) and causing the activation of PI3–Akt cascade with consequent increase of oxidative stress (27) and disruption of chemical communication.

The possibility that intracellular renin increases the intracellular calcium concentration through an increment of the inward calcium current as shown above exists because it is known that an increase of the intracellular calcium concentration can cause cell decoupling (19). However, it is not clear if the increment of the calcium concentration elicited by renin is enough to disrupt cell communication. Furthermore, the increase of inward calcium current caused by renin might be related to intracellular Ang II formation [see Ref. (22)] and recent studies indicated that intracellular Ang II disrupts chemical communication of heart cells (9).

An alternative explanation for the disruption of cell communication is an increment of oxidative stress induced by renin. Studies of Schefe et al. (21) indicated, for instance, that there is an intracellular renin receptor, which when activated by intracellular renin causes the translocation of a transcription factor (PLZF) from the cytoplasm to the nucleus and consequent activation of several genes including an enhanced transcription of the p85α subunit of the phosphatidylinositol-3 kinase (PI3K–p85α). Other studies (27) revealed that PI3K–Akt signaling activates production of ROS by opening of the mitochondrial ATP-sensitive K+ channel (mKATP channel). Is it possible that the activation of the intracellular renin receptor elicited by renin increases the oxidative stress through the PI3K–Akt signal pathway and reduces the gap junction permeability? Our results revealed that, indeed, intracellular renin enhanced oxidative stress in ventricular cardiomyocytes but it is not clear how much influential is the generation of reactive species on the inhibition of chemical communication described above.

Previous studies indicated that mitochondrial oxidative stress plays an important role in angiotensin II-induced gap junction remodeling (28). Because the binding site of the (pro)renin receptor is direct to the lumen of vesicles and to the extracellular space [see Ref. (1, 5, 29)], the binding of cytosolic renin to this receptor in the cytoplasm is unlikely unless the enzyme is taken up by vesicles or organelles and interact with the receptor synthesized locally inside these vesicles or organelles. The presence of the (pro) renin receptor in the nucleus (17) might support this view. Evidence is available that mitochondria is a site of aldosterone production and that overexpression of cytosolic renin increases aldosterone/renin ratio in transgenic animals (30). This finding plus the observation that aldosterone enhances the oxidative stress (31, 32) might indicate that the activation of the mineralocorticoid receptor, which is known to enhance the intracrine RAS (33), might be also involved in the decline of cell communication caused by intracellular renin as described above. Further studies will be needed to clarify this point.

Finally, a drastic fall of the intracellular pH, which is known to abolish cell communication (34), cannot be discarded. Although Ang II modulates the cardiac sodium/bicarbonate cotransporter with consequent decrease of intracellular pH [see Ref. (34, 35)], it is not known that if renin alone is able to cause intracellular acidification. Further studies will be needed to clarify this point.

The disruption of chemical communication elicited by intracellular renin is harmful for the heart function because it abolishes the cooperation among cardiac cells and prevents the exchange of amino acids, nucleotides, and other molecules between the cardiomyocytes. Moreover, the electrical uncoupling caused by the intracellular renin (2) contributes to the impairment of the synchronization of the electrical impulse and consequent generation of cardiac arrhythmias. On the other hand, evidence is available that renin and angiotensinogen mRNA are increased at the border of the infarct area (36, 37) raising the possibility that intracellular renin be of benefit preventing the spread of toxic metabolites and injury currents between damaged and non-damaged myocytes thereby avoiding the increment of the damaged area. According to this view, intracellular renin might be involved in the healing-over process, which is a fundamental aspect of heart cell biology (34). Further studies will be needed to clarify this point.

## Conclusion

(1) Intracellular renin disrupts the chemical communication between cardiac cells; (2) simultaneous administration of angiotensinogen enhanced the effect of intracellular renin; (3) enalaprilat reduces significantly the effect of renin supporting the notion that the formation of Ang II is involved in the decline of chemical communication; (4) aliskiren inhibited the effect of renin on chemical communication; (5) intracellular renin enhanced oxidative stress – an effect possibly related to Ang II formation; (6) a smaller direct effect of the enzyme on chemical communication cannot be discarded but the mechanism involved is not known. The effect of renin alone might be related to; (a) an increase of inward calcium current and consequent release of calcium inside the cell; (b) the activation of an intracellular renin receptor with consequent translocation of PLZF to the nucleus and increase of oxidative stress through the activation of PI3K–Akt pathway is an alternative explanation but requires its presence inside organelles or vesicle and that the (pro)renin receptor be synthesized locally; (c) a drastic fall of intracellular pH; (7) the present results indicate that intracellular renin due to internalization or *in situ* synthesis causes a severe impairment of chemical communication in the heart resulting in derangement of metabolic cooperation with serious consequences for heart function; (8) the harmful versus the possible beneficial effect of intracellular renin was discussed.

## Conflict of Interest Statement

The author declares that the research was conducted in the absence of any commercial or financial relationships that could be construed as a potential conflict of interest.
